# Supplementary material for: Strategies to enhance the level of service and safety of rural roads: A case study
Source: PLoS One. 2024 Mar 14;19(3):e0300525. doi: 10.1371/journal.pone.0300525 (PMC10939238; doi:10.1371/journal.pone.0300525)
Supplement: Attachment — Submitted filename: Response to Reviewers.doc [file pone.0300525.s001.doc]

**Original Manuscript ID:** PONE-D-23-25833

**Original Article Title: “**Enhancement strategies for Rural Roads management: a case study of Jintan, China”

**To:** PLOS ONE Editor

Dear Editor,

Thank you for giving us a chance to revise our paper.

Over the past month, we have spent great effort refining the manuscript, where the typing mistake, word usage, grammar error and sentence structure have been modified. Meanwhile, the comments raised by Reviewers and editors have got replied and implemented, together with the responses to Journal Requirements. Please see the separate response document uploaded in the system.

We are uploading (a) response document to the reviewers’ and editors’ comments (below), (b) a marked-up manuscript with indicated changes under the track changes mode in MS word, and (c) an unmarked manuscript without tracked changes.

Some necessary statements:

**Funder statement:** The funders had no role in study design, data collection and analysis, decision to publish, or preparation of the manuscript.

**Funding statement:**

"Local Innovation Talent Project of Yangzhou under Grant number 2022YXBS118.

Funder: Yangzhou Government, Jiangsu Province, China.

Recipient: Jun Zhang"

**Data availability:** The major data supporting the findings has already shown in the manuscript, and the other data including the GIS files of regional road network and the traffic characteristics data are available on request from the Chanzhou City Planning and Design Institute, email: [jsczghy@126.com](mailto:jsczghy@126.com).

Any further comments or requirements are welcome to us. Our deepest gratitude goes to you and the reviewers for your careful work. Thank you!

Best regards.

**Reviewer#1:** In general, the paper could use more editing because there are many places where the wording can be improved. The paper has no valuable contribution to the academic literature. The research methodology and data do not clearly support the suggested strategies.

**Author response:** Thanks for your comment. We have improved the wording in a more academic way. As to contributions, this paper focuses on the integration and application of multidisciplinary methods in the field of rural road transportation, and aims to introduce a systematic and feasible method from field investigation, data analysis (condition evaluation) and strategies proposal.

**Author action:** All suggested strategies from traffic safety inspection to intelligent system proposing are based on the investigated rural roads condition, used to better support the daily maintenance and management. In the beginning of *Current situation investigation* section, we have added some statements to clarify the data kinds, usage and the relationship between data and practical strategies. In the revised manuscript, the *Quantified traffic safety inspection* has been altered as a subsection under enhancement strategies.

**Reviewer#2, Comment #1:** The title of the paper is not communicating the content of the paper" The title can be modified as follows: Strategies to enhance the level of service and safety of a Rural Roads: A Case study.

**Author response:** Thanks for your comment!

**Author action:** We have modified the paper title according to your suggestion.

**Reviewer#2, Comment #2:** The literature is not organized properly. Example some where it is written as Tian et al. (2018) and also Griselda et al. pointed out that the completeness and correctness of markings or signs plays an important role in reducing crashes on rural highway [4]. please follow the consistency in writing the same. It is suggested that a good number of studies are available on pavement management system of rural roads or strategies and safety evaluation of rural roads. Further the authors referred many studies, but did not summarize the salient points or takeaway from the existing literature with respect to the current investigation. Also, the research gaps in the literature are missing.

**Author response:** Thanks for your valuable suggestions. There was some inconsistency in citing the reference during our writing, thanks for pointing out the problem. Meanwhile, additional literatures on pavement management and safety evaluation of rural roads have been added in the revised version. Literature summaries and research gap have also been supplemented.

**Author action:** The following 6 literatures on pavement management and safety evaluation of rural roads have been added in the revised manuscript, where relevant description or introduction are embedded. Note that in the updated version, the literatures have been reorganized from the perspectives of basic infrastructure improvement (including road alignment and pavement), traffic safety facilities configuration (including signs, markings, signals and etc.) and advanced management strategies (including the safety evaluation for infrastructure, and the intelligent traffic management strategies).

SR1 Nautiyal A, Sharma S. Scientific approach using AHP to prioritize low volume rural roads for pavement maintenance. Journal of Quality in Maintenance Engineering, 2022; 28(2): 411-429.

SR2 Guofeng Y, Haoyang W, Yuli P, Liping L. Research on application of the rural road performance assessment method in Jiangsu Province. Road Materials and Pavement Design, 2017; 18(sup3): 76-87.

SR3 Pasindu HR, Ranawaka RKTK, Sandamal RMK, Dias TWKIM. Incorporating road safety into rural road network pavement management, International Journal of Pavement Engineering, 2022; 23(12): 4306-4319.

SR4 Zhang M, Gao S. Evaluation of Rural Road Asphalt Pavement Performance Based on Matter Element Analysis. 2nd International Conference on Civil Engineering and Rock Engineering (ICCERE), 2017.

SR5 Francisco JCT, Ana MPZ, Manuel CU, Alfredo GG. New geometric design consistency model based on operating speed profiles for road safety evaluation, Accident Analysis & Prevention, 2013; 61: 33-42.

SR6 Vaiana R, Perri G, Iuele T, Gallelli V. A comprehensive approach combining regulatory procedures and accident data analysis for road safety management based on the European Directive 2019/1936/EC. Safety, 2021; 7(6): 1-24.

According to your advice, the authors have supplemented a brief summary after each research perspective in order to illustrate the salient points. The summaries of three reorganized research perspectives are listed as follows.

*Basic infrastructure improvement:* Current literatures on the enhancement of basic infrastructure for rural roads are safety-based geometric design and pavement management, with common considerations on traffic volume, traffic composition and design speed, and the results can provide important references for enhancement strategies.

*Traffic safety facilities configuration:* It can be concluded that the road alignment and traffic environment are key basis for the configuration of safety facilities on rural roads. The configuration schemes can be improved to the level above standards by analyzing the interaction mechanisms among facility installation parameters, drivers’ perception attributes and driving behaviors.

*Advanced management strategies:* Generally, existing strategies have covered the life-cycle management of rural roads including planning, construction, operation and maintenance, but the strategies are independent from each other, the integration of different monitoring and measuring data sources appears to be the development trend for smart management and decision.

The research gap mentioned in the original manuscript is how to evaluate the status of different rural roads effectively and adopt the appropriate enhancement strategies correctly considering the feasibility. In the revised manuscript, we have modified the research gap by adding the following statement:

Most existing literatures focus on presenting a specific method or strategy to improve the level of service or safety, it is undeniable that the proposed strategies are theoretically feasible and useful, but the methods of different studies are usually mutual separated to each other, causing the problem of global evaluation and enhancement when it comes a rural network. Meanwhile, most theories are difficult to be utilized in the rural area due to the limitation of financial support and the lack of professional engineers for rural roads.

**Reviewer#2, Comment #3:** The Table 1 titled with "Basic information of major road sections" i think this is road inventory information!! What do you mean by single or single +triple or dual!! The author is suggested to quantify the same. Some where it is mentioned in the manuscript that single lane means (10m), is there any reference for this!! All around the world including the developed countries have an 3.75 m as single lane width of the road!! please clarify on the same. !! Similarly what is triple lane?

**Author response:** Thanks for your comment. We have noticed this kind of ambiguous expression during the writing of original manuscript. As you can see in the table, this column corresponds to the carriageway attribute, where “single” means that the cross-section type is single carriageway road, and “single+ triple” means that part of the road cross-section is single carriageway type and part is the triple carriageway type. Please note that **the carriageway isn’t equal the conception of lane**, where the number of carriageways is decided by the separation infrastructures like the central green belt and the isolation barriers. As indicated in Fig.8 in the revised manuscript (original Fig.7), the cross-section type of Jianshe Road is single carriageway with a total breadth of **10m**, **composed of two 3.5m width motor lanes and two 1.5m width non-motor lanes**.

**Author action:** To make this kind of expression more understandable, the original column of carriageway has been modified as the cross-section type, by number 1 to 4, where the explanation of the cross-section type number has been added as a new table in the revised manuscript.

TableS1. Illustrations for typical rural road cross-section type

| Type No. | Cross-section type | Illustration | Characteristic |
| --- | --- | --- | --- |
| 1 | Single carriageway |  | without central separations;  mixed traffic flow and conflict |
| 2 | Dual carriageway |  | with central separations;  without directional conflict;  with lateral conflict between motor and non-motor traffic |
| 3 | Triple carriageway |  | with two lateral separations;  without motor and non-motor conflict |
| 4 | Mixed | Single carriageway + Dual carriageway  Single carriageway + Triple carriageway | The main road is single carriageway, the multi-carriageway is applied in the road sections across the town area to improve the traffic safety. |

As to your concern that the developed countries have a 3.75 m as single lane width, this kind of lane width is not commonly applied in the design of most Chinese rural roads, due to their lower standard level. According to the rule 6.2.1 from the Specifications for Highway Geometric Design issued by ministry of transport of the People’s Republic of China, shown in Figure S1 below. In our study area, most rural roads own a lane width of 3.5m, because the top speed limit is almost 60 km/h (see Fig.6 in the manuscript), while some own a lane width of 3.75m under an 80 km/h design speed.

**Figure S1. Lane width criteria in the highway design standard of China**

**Reviewer#2, Comment #4:** Authors are suggested to include the road inventory data on such as length of road, width of road, existing pavement cross section details, land use, land pattern, rainfall and traffic data etc.!!

**Author response:** Thanks for your comment.

**Author action:** We have modified Table 1 in the manuscript with additional information of length, traffic data, surrounding land use, as shown in Table S2 below. As to the data like rainfall mentioned in your comment, all roads in the study area share a same climate condition. We have added a statement of climate in the revised version: The average daily rainfall is about 2.91 mm, where days with rainfall over 10 mm only accounts for 2.85% of the whole year. Meanwhile, the average wind speed is about 3.3 m/s, and the average daily sunshine percentage is about 46%.

We have also added a map of regional rural roads network, as shown in Figure S2 below (Fig.2 in the revised manuscript), in order to increase the readability of *Roads Information* section.

**Table S2**. Basic information of major road sections

| **Road** | **Section** | **Length**  **（km）** | **Cross-section parameters** | | **Traffic** | | **Major land use** |
| --- | --- | --- | --- | --- | --- | --- | --- |
|  |  |  | **Type** | **Lane distribution and surface pavement** | **Volume (PCU/day)** | **Truck**  **ratio** |  |
| X301 | JSH-X203 | 12.79 | 1 | Motor: 3.5m×2  Non-motor: 1.5m×2  (Asphalt) | 1130 | 6.3% | Agriculture |
|  | X203-X102 | 5.04 | 1 |  | 610 | 4.7% |  |
| X302 | JSH-X202 | 8.38 | 1 | Motor: 3.5m×2  Non-motor: 1.5m×2  (Asphalt) | 1560 | 8.1% | Agriculture |
|  | X202-JZ | 1.19 | 1 |  | 2410 | 13.6% |  |
|  | JZ-X203 | 2.85 | 1 |  | 2050 | 15.1% |  |
|  | X203 west | 4.89 | 1 |  | 1520 | 12.4% |  |
| X303 | JSH-X202 | 3.94 | 1 | Motor: 3.5m×2  Non-motor: 1.5m×2  (Asphalt) | 2170 | 7.5% | Industry |
|  | X202-XYQ | 1.11 | 1 |  | 1620 | 8.4% | Residence |
|  | XYQ-X203 | 3.79 | 1 |  | 1540 | 8.2% | Residence |
|  | X203 west | 3.99 | 1 |  | 640 | 6.2% | Agriculture |
| Jinzhuang (JZ) | X301-X302 | 3.85 | 4 | Motor: 3.5m×2  Non-motor: 1.5m×2  +  Motor: 3.5m×2  Lateral barriers: 0.5m×2  Non-motor: 2m×2  (Asphalt) | 1350 | 2.1% | Residence |
| Xiangyangqiao (XYQ) | X303-X203 | 4.87 | 2 | Motor: 3.5m×2  Non-motor: 1.5m×2  (Asphalt) | 780 | 3.6% | Residence and commercial |
| Jincheng  (JC) | JSH-BT | 1.48 | 2 | Mortor:3.75m×2  Non-Motor: 1.5m×2  (Cement) | 1230 | 24.5% | Industry and warehouse |
|  | BT-NDY | 0.88 | 2 |  | 1510 | 27.7% |  |
|  | NDY-NHY | 1.62 | 2 |  | 1670 | 31.6% |  |
|  | NHY-NJH | 1.71 | 2 |  | 1380 | 32.1% |  |
| … | … |  | … |  |  |  |  |
| X204 | X302 north | 1.51 | 1 | Mortor:3.5m×2  Non-Motor: 1.5m×2 (Asphalt) | 560 | 4.1% | Agriculture |
|  | X302-S265 | 1.78 | 1 |  | 660 | 4.7% | Residence |
|  | S265-X303 | 3.11 | 1 |  | 590 | 6.2% | Industry and agriculture |
|  | X303 south | 7.84 | 1 |  | 630 | 5.4% |  |
| Jinsha  (JSH) | JC south | 1.57 | 2 | Mortor:3.75m×4  Central green belt:2m  Non-Motor: 1.5m×2  (Asphalt) | 4310 | 5.9% | Industry and agriculture |
|  | JC-NJH | 5.66 | 2 |  | 4200 | 5.5% |  |
|  | NJH-X301 | 1.73 | 2 |  | 3780 | 6.4 % |  |

**Figure S2. Rural roads network within the study area**

**Reviewer#2, Comment #5:** Further, the authors has done a field study and made the observations on the sign boards and other facilities visually! Rather mentioning that the color was faded, the sign boards are not visible and they are not properly protected.....they should have done through quantification in terms of their size, shape and reflectivity as per some standard and the results would have been presented!! for example it is mentioned that " Due to the branch ways connecting villages and current road, yield signs have been set on the corner of the branch way. No-parking signs are also laid along the busy town sections" are there any standards or guide lines available in your country for installation of these sign boards!! please take care about the same.

**Author response:** Thanks for your comment. All our inspection on traffic signs is based on the guidelines from both national standards and industry standard. Considering the article length, the part of regulations and standards for traffic signs is ignored in the original manuscript, which we thought will affect the readability. However, according to your suggestion, the authors now think that it is necessary to briefly state the standards we used.

**Author action:** In the beginning of Traffic facility configuration section, we have added a subsection of *Adopted standards and* *guidelines* to indicate the criteria for facility configuration analysis. The inspection on traffic signs are based on the following two standards, one is the Road Traffic Signs and Markings (GB5768.2-2022) and the Road Traffic Sign Panels (JT/T 279-2004), as shown in the Figure S3 below.

**Figure S3. The standards used during the inspection of traffic signs**

Generally, the adopted standards are summarized from the following three perspectives according to our study focus.

(1) Quantified standards for sign size and appearance

The size of sign panels should conform to the related standards in GB5768.2, where the acceptable size deviation is ± 5mm for normal panels, and ± 0.5% for panels longer than 1.2m.

The surface of sign panels should be flat with an unevenness less than 3mm/m.

The following defects are not allowed on the traffic sign panels: Cracks, wrinkles and peeling-off edges; Obvious scratches, damages and the non-uniform color painting; Blisters or bubbles larger than 10 mm2 on any surface of a 50cm×50cm size; The uneven retroreflective performance.

The size of sign panels depends on the roads design speed. The design speed of rural roads usually ranges from 40 km/h to 70km/h, and the corresponding sizes of different prohibition and directional size shapes are listed in Table S3 below.

**Table S3. Standard shape sizes for prohibition signs and directional signs in rural area**

| **Shape type** | | **Parameters** | **Size value (cm)** |
| --- | --- | --- | --- |
| Octagonal sign | | Outside diameter | 80~100 |
| Triangular sign | | Side length | 90~110 |
| Circular sign | | Outside diameter | 80 |
| Rectangle sign | Off-limits or restricted area sign | Height×Width | 170×120 |
|  | One-way traffic sign | Height×Width | 80×40 |
|  | Square sign | Outside diameter | 80 |
|  | Other rectangle sign | Height×Width | 80×64 |

(2) Requirements for sign angles

The installation angle should avoid the glare to drivers from the sign panel.

The roadside traffic signs should be vertical to the road centerline or at a certain angle from the vertical direction. Specifically, the angle of prohibition signs and directional signs should be 0~10° or 30~45°, the angle of warning signs and guiding signs should be 0~10°. As indicated in the figure below.

Figure S3. The requirements for the angles of specific traffic signs

(3) Regulations for sign locations

Due to the large number of T-type crossings or non-signalized crossings, special focuses are paid on the yielding signs and branch way posts.

The stop yielding signs should be installed on the mouth of branch way under the following conditions: the left-turn motor traffic needs control, or the conflict between motor traffic and non-motor traffic needs control; the crossing sight distance is limited or the intersection is a hazardous location; the signal intersection without all day 24-hour control.

As compared to the stop yielding signs, the speed reduction yielding signs can be installed at the mouth of branch way with a better sight distance or a lower accident risk, or at the entrances of non-signal roundabouts.

The branch way posts should be installed on the two sides of branch mouth, in order to warn the drivers along the main road against the potential conflict with vehicles from the branch way. The location and feature of branch way posts are illustrated in the figure below.

Figure S4. The installation standards of branch way posts

As to the sign reflectivity raised in your comment, we didn’t measure the sign retroreflection coefficients due to the lack of professional measuring devices during the field investigation. Therefore, we can only judge the reflection performance by the fading condition of sign color. We hope for your understanding, because investigating the facility status and distribution of rural roads within the area of about 350 km^2^ (shown in Figure S2 in the response to Reviewer#2, Comment #4) was already a tough work for us.

Thanks again for the valuable advice. We have modified the description of current sign borads condition in a more quantified way.

**Reviewer#2, Comment #6:** The equation (1) AND (2) the complete notations are missing !! Table 2 what are the units of length(m/km/cm/mm)???. What do you mean by complex network??

**Author response:** Thanks for your comment. First of all, we want to explain the usage of Complex Network analysis. The complex network theory is usually used to study the geometric properties and structural characteristics, and here in our paper it is used to study the roadnet connectivity, in order to: (1) figure out the topological feature of studied roadnet, and (2) analyze the inherent road accessibility within the rural area. The results of complex network analysis will support the evaluation of level of service and safety. E.g., the result of degree centrality shows that there exist many T-type or Y-type crossings in the area, which will be conflict locations without high-standard safety-related facilities or signal control equipment.

As to the units of shortest path length, it is a dimensionless parameter in the context of a complex network. In the complex network, the length of one edge connecting adjacent nodes is 1, and one path is composed of several edges. In other words, the length of a path refers to the number of edges along the path. As described before Equation (2), “Note that the length in the complex network is not equal to the actual road length, the length of an edge between nodes is counted as 1”.

**Author action:** The notations of Equation (1) and (2) have been completed, together with a modified explanation of path length. We have also added a literature on complex network:

SR7: Soh H, Lim S, Zhang T, et al. Weighted complex network analysis of travel routes on the Singapore public transportation system. Physica A: Statistical Mechanics and its Applications, 2010, 389(24), 5852-5863.

**Reviewer#2, Comment #7:** Overall not much novelty and in the paper. However the conclusions are very generic and can be re written with respect to the work. the pictures and photographs are not good quality!

**Author response:** Thanks for your valuable suggestion in highlighting the contribution of current work in the *Conclusion* section.

As to the picture display quality, all figures in our manuscript are TIF images with the resolution over 250 DPI (Dots Per Inch). We have downloaded the review version and made a comparison. It is found that the pictures exported by the submission system have lost some quality, as you can see in the figure below. The original picture files uploaded in the submission are original photographs with good quality.

|  |  |
| --- | --- |
| **(a) Pictures exported in submission system** | **(a) Original pictures with high resolution** |

**Figure S5. Comparison between system exported pictures and our original pictures**

During your review procedure, if you want to check the original pictures, you can click the linkage in the upper-right corner to download them, as shown in the figure below. Thank you!

Figure S6. Downloading link on the pages of review version

**Author action:** We have rewritten the conclusion with more findings and contributions from current work, so that the conclusion wouldn’t seem too general. Please see the *Conclusion* section in the revised manuscript. The following statements have been added, and other description have got modified together.

The field study reveals that the common problems of rural roads are safety infrastructure maintenance and traffic operation management, arriving from the outdated construction standards and the long-term absence of effective supervision.

During the analysis and evaluation on rural roads, it is found that there exists a strong relation between the traffic characteristics and the level of service and safety, e.g., the higher proportion of trucks and lorries usually corresponds to a worse pavement condition, a worse signs and markings visibility and a higher traffic conflict rate. Therefore, it is of great urgency to establish the intelligent management system for rural roads, with embedded technologies of multi-source data analysis, risk visualization and assistant decision.

**Reviewer#2, Comment #8:** It requires a lot of language corrections and improvements.

**Author response:** Thanks for your comment.

**Author action:** Under the help of a colleague with overseas education experience, as well as the MS Office plug-ins like Ginger writer and style writer, the word usage, the grammar and the sentence structure have got improved in the revised version.

**Editors, Keypoint #1:** Revise the title to better reflect the content of the paper, aligning it with the strategies to enhance the level of service and safety of rural roads discussed in the study.

**Author response:** Thank you!

**Author action:** The title has been revised. Please see the response to Reviewer#2, Comment #1.

**Editors, Keypoint #2:** Reorganize the literature review, summarizing the relevant points from existing studies and identifying research gaps.

**Author response:** Thanks for your suggestion!

**Author action:** In the revised manuscript, we have reorganized the literature review part by emphasizing the major contribution and conclusion of related literatures, where some literatures have been replaced and some new literatures have been added, and the research gap of current studies have been enriched. Please see the response to Reviewer#2, Comment #2.

**Editors, Keypoint #3:** Quantify and provide references for terms and information related to road sections, such as single lane, triple lane, and dimensions, ensuring consistency and clarity.

**Author response:** Thank you!

**Author action:** The ambiguous terms have been explained and modified with detailed figure illustrations and guidelines. Please see the response to Reviewer#2, Comment #3.

**Editors, Keypoint #4:** Improve the presentation of field study observations by quantifying and providing standardized measurements, such as size, shape, and reflectivity, according to applicable guidelines or standards.

**Author response:** Thank you!

**Author action:** The quantified applicable standards and guidelines of sign size, shape and location used in the field observations have been added in the revised manuscript. Please see the response to Reviewer#2, Comment #5.

**Editors, Keypoint #5:** Address notational completeness, providing clear explanations and references for equations and tables.

**Author response:** Thank you!

**Author action:** Please see the response to Reviewer#2, Comment #6. The notations of variables and parameters in every equation have been checked.

**Editors, Keypoint #6:** Clarify units of measurement in tables, ensuring consistency and accuracy.

**Author response:** Thanks for your valuable comment.

**Author action:** Please see the explanation of unit problem of shortest path in the response to Reviewer#2, Comment #6. The units of other quantified indicators in other tables have been clarified, and the consistency and accuracy have also been clarified and ensured.

**Editors, Keypoint #7:** Strengthen the conclusions by highlighting the novel aspects of your work and its contribution to the academic literature.

**Author response:** Thank you!

**Author action:** Please see the response to Reviewer#2, Comment #7.

**Editors, Keypoint #8:** Enhance the quality of visuals, such as pictures and charts, ensuring they are of sufficient resolution and clarity.

**Author response:** Thank you! All pictures are of high resolution, while the exported pictures in the PDF document for reviewers are distorted, making them look blurry.

**Author action:** Please see the response to Reviewer#2, Comment #7.

**Editors, Keypoint #9:** Clearly explain the research methodology employed in your study, including the data collection methods, sample selection, and any statistical analyses performed. Justify how this methodology supports the strategies proposed.

**Author response:** Thanks for your valuable advice.

**Author action:** The data collection methods has been added as an independent subsection named under the *Current situation investigation section*, together with the investigation content. The data collection methods applied in the field observations and measurement include:

- Photographing method. Taking photos of roadside environment, road pavement and traffic-related facilities. The photos are used as basic references to illustrate current condition.
- Aerial video recording. Recording the traffic status of rural road sections and signal-control intersections with UAVs (unmanned aerial vehicle). The aerial videos are the basis of road traffic analysis, including traffic volume, traffic density and conflicts distribution.
- Positioning method. Marking the positions of inspected infrastructures, facilities and signs via GIS map tools, which synchronizes with the photographing method.
- Trajectory recording method. Recording the trajectory data during the repeated paths driving test, which are used in the speed distribution analysis and travel time estimation. The trajectory data are also helpful in both trucks dynamic path management and reconstruction sections traffic guidance.
- Physical Measurement. Measuring the physical parameters of road infrastructures and signs by tape measures, such as the lane width, the sign size & location, the guardrail length and etc. The measurement data are key basis of traffic facilities safety check and evaluation.
- History data investigation. Obtaining the data like area population distribution, road maintenance records, road accidents record from the published data of local government and transportation sectors. These kinds of data can be applied in indicators calculation and prediction, especially for the decision making in traffic control and infrastructure maintenance.

The relationship between the methodology and the strategies are strengthened.

**Editors, Keypoint #10:** Provide a detailed explanation of the data used in your study, including its sources, relevance, and reliability. Highlight how this data supports and strengthens the suggested strategies.

**Author response:** Thank you! This suggestion is similar to the previous suggestion.

**Author action:** The data we collected are important to support subsequent condition analysis and strategy proposal. The usage of each data has been added in the revised manuscript.

**Editors, Keypoint #11:** Consider any limitations or potential biases in your research methodology or data and discuss how these were addressed or mitigated.

**Author response:** Thank you!

**Author action:** Some statements have been added in the revised version. E.g., the standards and guidelines during facility inspection are only applicable in China, since the transportation condition of rural roads varies in different areas. Some strategy can only be implemented by the combined efforts of relevant administrators and managers.

**Journal requirement #1:** Please ensure that your manuscript meets PLOS ONE's style requirements, including those for file naming.

**Author response:** Thank you! The style and format of manuscript and files are correct.

**Journal requirement #2:** In your Methods section, please provide additional information regarding the permits you obtained for the work. Please ensure you have included the full name of the authority that approved the field site access and, if no permits were required, a brief statement explaining why.

**Author response:** Thank you! The field investigation work was approved by the Science and Technology Bureau of Changzhou. We have provided this in the revised manuscript.

**Journal requirement #3:** We suggest you thoroughly copyedit your manuscript for language usage, spelling, and grammar.

**Author response:** Thank you! Please see the response to Reviewer#2, Comment #8:

**Journal requirement #4:** Please ensure that you include a title page within your main document.

**Author response:** Thank you! Now the title page has become a part of the manuscript file.

**Journal requirement #5:** Please remove any funding-related text from the manuscript and let us know how you would like to update your Funding Statement. Please include your amended statements within your cover letter; we will change the online submission form on your behalf.

**Author response:** Thank you! The funding-related text has been removed from the manuscript. The funding statement is:

"Local Innovation Talent Project of Yangzhou under Grant number 2022YXBS118.

Funder: Yangzhou Government, Jiangsu Province, China.

Recipient: Jun Zhang"

**Journal requirement #6:** Please state what role the funders took in the study.

**Author response:** Thank you! Funder statement: The funders had no role in study design, data collection and analysis, decision to publish, or preparation of the manuscript.

**Journal requirement #7:** PLOS only allows data to be available upon request if there are legal or ethical restrictions on sharing data publicly.

**Author response:** Thank you! The major data supporting the findings has already shown in the manuscript, and the other data like GIS files of road network and traffic characteristics a are available on request from the Chanzhou City Planning and Design Institute (email: jsczghy@126.com), the data are not publicly available due to privacy restrictions.

**Journal requirement #8/9:** The copyright problem of some figures.

**Author response:** Thank you! All figures in the manuscript are originally drawn by the authors, where some regional satellite maps used as backgrounds in Figures 1,5,6,7 and 14 are free for users to use, and other contour lines, markers, characters and density heat map layers are completely plot by the authors. The background map images we use are from the Chinese National Platform for Common Geospatial Information Services (public domain) (<https://www.tianditu.gov.cn/>), as shown below. **We have deleted the satellite maps in the original figures, and replaced them with newly self-drawn illustrations**.

**Figure S7. Map images source used in our study.**

In Figures 2-4, most pictures are photos taken by authors Jun Zhang and Qiannan Ai during the field investigation, other pictures have been replaced with our own pictures in the revised version. The replacements are shown below. All of the authors approve to publish these figures specifically under the CC BY 4.0 license.

**Figure S8. Pictures replaced with our own photographs.**

**Editors further request #1:** Please explain where the authors obtained the map image in the “Topography” section of Figure 1.

**Author response:** Thank you! The image of Hills & slopes in Fig 1 represented by contour line was plot by the authors based on the elevation data encoded in DEM format, as shown in the figures below, where the background map in Figure S10 is obtained from the Chinese National Platform for Common Geospatial Information Services (public domain) (https://www.tianditu.gov.cn/), free for public use, as we explained in page 14 of the Response to Reviewers document.

**Figure S9. The elevation data encoded in DEM format.**

**Figure S10. The self-drawn contour lines in a GIS analysis tool upon the data in Fig S9.**

**Editors further request #2:** Please explain where the authors obtained the map image in Figure 2.

**Author response:** Thank you! Fig 2 is drawn by the authors using the TransCAD software, which is a professional tool in the field of transportation planning and management, as indicated in the figure below.

**Figure S11. The regional road network drawn by the authors in the TransCAD software.**

**Editors further request #3:** Please note that Figures 6, 7, and 8 include images labeled “Cross-Section Parameters” that contain watermarks.

**Author response:** The cross-section images with watermarks in Figs 6-8 have been updated. Thanks for reminding us of this problem. Original pictures with watermarks were generated in the trial version installed in the personal laptop, and the updated pictures are generated by the official version installed in the laboratory computer, as shown in the figure below.

**Figure S12. Updated cross-section images plotting by official registered software JASOFT.**

**Editors further request #4:** Please explain where the authors obtained the two map images in Figure 17.

**Author response:** The two map images in Fig 17 indicating the global risk distribution and flow distribution are also based on our self-drawn roadnet in TransCAD, which is identical to the basic roadnet shown in Fig 2. As to the global risk distribution image in Fig 17, the heat map of potential risk severity was output by ArcGIS, based on the evaluation results of road section risk and intersection risk. The flow/noise/emission distribution image in Fig 17 was totally output by TransCAD through its inherent drawing tools, based on the bi-directional flow/emission data of every road sections. However, the background map picture in the flow/noise/emission distribution picture has been removed in the newly updated figure file.

**Figure S13. The self-drawn flow distribution in the TransCAD software based on investigated volume**

**Editors further request #5:** Please explain where the authors obtained all the images in Figures 6, 7, 8, and 17.

**Author response:** The other images in Figs 6-8 are all photographed by the authors during the field investigation, as mentioned in the Current situation investigation section in the manuscript. The other images in Fig 17 are all owned by the authors, shared with the authors other research projects in smart road maintenance and traffic condition diagnosis.

**Editors further request #6:** Please state whether any of the images in Figures 1, 2, 6, 7, 8, and 17 have been previously copyrighted.

**Author response:** The authors declare that none of the images in Figs 1,2,6,7,8 and 17 has been previously copyrighted.
